# Supercharged penguins—supplementary feeding reshapes tawaki/Fiordland penguin winter migration

**DOI:** 10.7717/peerj.21630

**Published:** 2026-08-10

**Authors:** Owen Dabkowski, Thomas Mattern, Klemens Pütz, Richard Seed, Benno Lüthi, Ursula Ellenberg

**Affiliations:** 1 Department of Marine Science, University of Otago, Dunedin, New Zealand; 2 The Tawaki Trust, Dunedin, New Zealand; 3 Global Penguin Society, Marcos Zar, Puerto Madryn, Chubut, Argentina; 4 Department of Zoology, University of Otago, Dunedin, New Zealand; 5 Antarctic Reseach Trust, Am Oste-Hamme-Kanal, Bremervörde, Germany; 6 Antarctic Research Trust, c/o Zoo Zürich, Zürichbergstr, Zürich, Switzerland

**Keywords:** Penguins, Satellite tracking, Telemetry, Winter migration, New Zealand, Tawaki, Fiordland penguin, Conservation, Rehabilitation, Supplementary feeding

## Abstract

Migration in a species is primarily a response to resource availability, shelter, thermoregulation, predator avoidance, or seasonal environmental change. Tawaki/Fiordland penguins (*Eudyptes pachyrhynchus*) have earned the nickname “marathon penguins” as they migrate 2,500 km from their nest sites during the short period between breeding and annual moult, despite productive waters being found around their breeding sites in southern New Zealand. A similar directional tendency is evident during the subsequent winter migration, during which birds target subantarctic waters south of Tasmania, often reaching the polar front. However, during a previous study, two birds were tracked that not only moulted outside the species’ breeding range on the east coast of New Zealand’s South Island, but did so in the care of a rehabilitation centre. In care, birds are generally fed throughout the moult even though penguins cannot forage during the moult as all feathers are replaced simultaneously. Although these two birds ceased transmitting within days of release, they appeared to follow different trajectories—south and south-east—compared to wild birds that consistently travel west and south-west. The apparently aberrant behaviour of these east coast birds raised the question of whether moulting location and/or supplementary feeding during rehabilitation could affect subsequent winter migration. To examine this, between February and March 2020 we deployed satellite transmitters on 18 tawaki at the conclusion of their annual moult, comparing three groups: birds moulting on the west coast, birds moulting unassisted on the east coast, and birds released from rehabilitation facilities following supplementary feeding during the moult. West and east coast tawaki followed broadly similar routes—south-westward and southward respectively—reaching subantarctic waters south of Tasmania or south of mainland New Zealand. Supplementarily fed birds departed heavier than either wild group, ranged more widely and farther, resulting in longer trips and less focused movement patterns. Migratory differences among groups were driven principally by departure body condition, suggesting that supplementary feeding during the moult may substantially alter migratory behaviour and affect their subsequent annual life history. As such, this study provides an evidence-based foundation for refining rehabilitation protocols for penguin species undergoing moult in human care.

## Introduction

For most species, migration is primarily a response to resource availability, shelter, thermoregulation, predator avoidance, or seasonal environmental change (Furey et al., 2018; Bonnet-Lebrun et al., 2021; Webber & McGuire, 2022). Three broad mechanistic classes have been proposed to underlie migratory behaviour: non-orientated movement, oriented movement, and memory mechanisms (Mueller & Fagan, 2008). Non-orientated movement, such as diffusion or kinesis, involves displacement in largely random directions in response to local stimuli (Mueller & Fagan, 2008). Oriented movement occurs over greater distances and is directed by distant cues, often producing predictable trajectories toward or away from a stimulus (Mueller, Fagan & Grimm, 2011). Memory-based movement draws on previously acquired spatial information, whether from individual experience, social transmission, or genetic inheritance (Winter & Stich, 2005; Mueller & Fagan, 2008; Orgeret et al., 2019; Falcón-Cortés et al., 2023).

Tawaki/Fiordland penguins (*Eudyptes pachyrhynchus*, referred to as ‘tawaki’ hereafter) earned the nickname ‘marathon penguins’ owing to their extensive pre-moult migrations. During these migrations tawaki travel up to 2,500 km from their breeding colonies to forage at subantarctic frontal systems, covering total swimming distances of up to 6,800 km in just 8–10 weeks before returning to moult (Mattern et al., 2018). What makes tawaki’s pre-moult migration particularly puzzling is that it appears ecologically counterintuitive. Rather than exploiting the high marine productivity around mainland New Zealand that peaks in summer, tawaki expend enormous energy reserves travelling thousands of kilometers to the subantarctic, only to replenish their body condition through hyperphagia in waters closer to the mainland on the return journey (White, 2024). In terms of net energetic gain, the journey seems difficult to justify by conventional cost-benefit logic. This may reflect a deeply conserved migratory tendency inherited from *Eudyptid* ancestors that evolved in the subantarctic (Cole et al., 2019), where seasonal changes in productivity make long-distance migration an adaptive necessity (Croxall & Davis, 1999). For tawaki, which breed in the temperate, productive waters of mainland Aotearoa New Zealand, this ancestral drive may persist in the absence of the ecological pressures that originally shaped it (Mattern et al., 2018). This interpretation is further supported by the species’ post-moult winter migration (Mattern et al., 2025).

Unlike other crested penguins, which typically disperse laterally from their breeding sites during winter, tawaki cross multiple water masses and presumably variable foraging environments, consistently reaching the Polar Frontal Zone south of Tasmania (Mattern et al., 2025). Other crested penguins instead remain within a consistent water mass or track along a single frontal zone to maintain favourable foraging conditions (Green, 2023). Broadly consistent with the pre-moult dispersal, this winter migration follows a similar southwest trajectory, though without the same energetic time constraint (Mattern et al., 2018; Mattern et al., 2025). While most tawaki moult at west coast sites within their core breeding range, the species is also regularly encountered moulting along the eastern South Island, on the coasts of Southland, Otago, and South Canterbury, during February and March (Mattern & Wilson, 2019).

West and east coast moulting sites sit in markedly different current regimes. Along the west coast, the Fiordland Current flows predominantly south (Chandler, Melissa & Smith, 2021; Stevens et al., 2021), broadly aligned with the southwest trajectory tawaki follow at the beginning of both pre-moult and winter migration (Mattern et al., 2018; Mattern et al., 2025). Along the east coast, the Southland Current instead flows north, opposing rather than reinforcing the penguins’ southward departure, and the Subtropical Front sits close against and parallel to the coast rather than standing offshore (Sutton, 2003; Stevens et al., 2021). East and west coast birds therefore depart into oceanographically distinct settings, providing grounds to expect trajectory and space-use differences.

During the 2019 winter tracking study, two tawaki were opportunistically fitted with transmitters after completing their moult at a rehabilitation facility on the east coast of the South Island (Mattern et al., 2025), where they were both supplementarily fed through the moult (Oamaru Penguin Colony, pers. comm., 2019). One bird lost its device shortly after release, but the other tracked southeast. This was a striking deviation from the southwest trajectory observed in all west coast birds, and toward one of the least productive marine areas in the New Zealand region (Murphy et al., 2001). While this observation was limited to a single individual, its stark departure from previously established migration trajectories combined with the vastly different oceanographic conditions of the east coast, suggested that this might not have been merely an isolated anomaly, but potentially indicative of a broader, undocumented population-level pattern. Furthermore, because of supplementary feeding while in care, the aberrant trajectory could also have been an artifact of human intervention. This raised the question of whether east coast birds follow a fundamentally different migratory trajectory, and whether rehabilitation itself may alter or disrupt normal migratory behaviour.

The objectives of this study were therefore to:

(A) examine and compare the post-moult migratory patterns of three groups of tawaki: penguins moulting on the west coast, birds moulting unassisted (*i.e.*, in the wild or unfed in rehabilitation) on the east coast, and birds released from rehabilitation facilities who received supplementary feeding on the east coast;
(B) identify potential drivers of any observed migratory differences—in particular, whether any divergent trajectories reflect the birds’ geographic starting point, the physiological and behavioural aftereffects of rehabilitation, or some combination of both; and
(C) assess the implications of these findings for current rehabilitation practices for the species.

## Methods

### Study species & sites

Tawaki are the only crested penguin species to breed on a continental landmass. Their colonies are distributed from Bruce Bay on the central West Coast of Aotearoa New Zealand’s South Island southwards through Fiordland and Rakiura/Stewart Island. Occasional breeding attempts have been reported from the east coast (Young, Pullar & McKinlay, 2015), but no established colonies exist there. Nevertheless, tawaki are regularly encountered along the eastern South Island during February and March, when some return to moult on the coasts of Southland, Otago, and South Canterbury (Mattern & Wilson, 2019).

Following the breeding season, which concludes in mid-November to early December, tawaki undertake a pre-moult migration, generally to the Subantarctic and Polar Front regions south of Australia (Mattern et al., 2018). They return to the New Zealand mainland in January and February to complete the annual moult (Mattern & Wilson, 2019). Birds moulting on accessible eastern beaches and waterfronts subject to significant human visitation are often uplifted and transferred to rehabilitation facilities for their own safety, while those in more remote sites are usually left undisturbed.

For the purposes of this study, satellite transmitters were deployed on tawaki assigned to one of three groups based on moulting location and feeding regime during the moult:

• west coast birds (moulting at a Fiordland site within the core breeding range),
• east coast birds (moulting in the wild or unfed in rehabilitation along the eastern South Island), and
• supplementarily fed birds (moulting at rehabilitation facilities on the east coast where hand-feeding was provided throughout).

**Figure 1:**
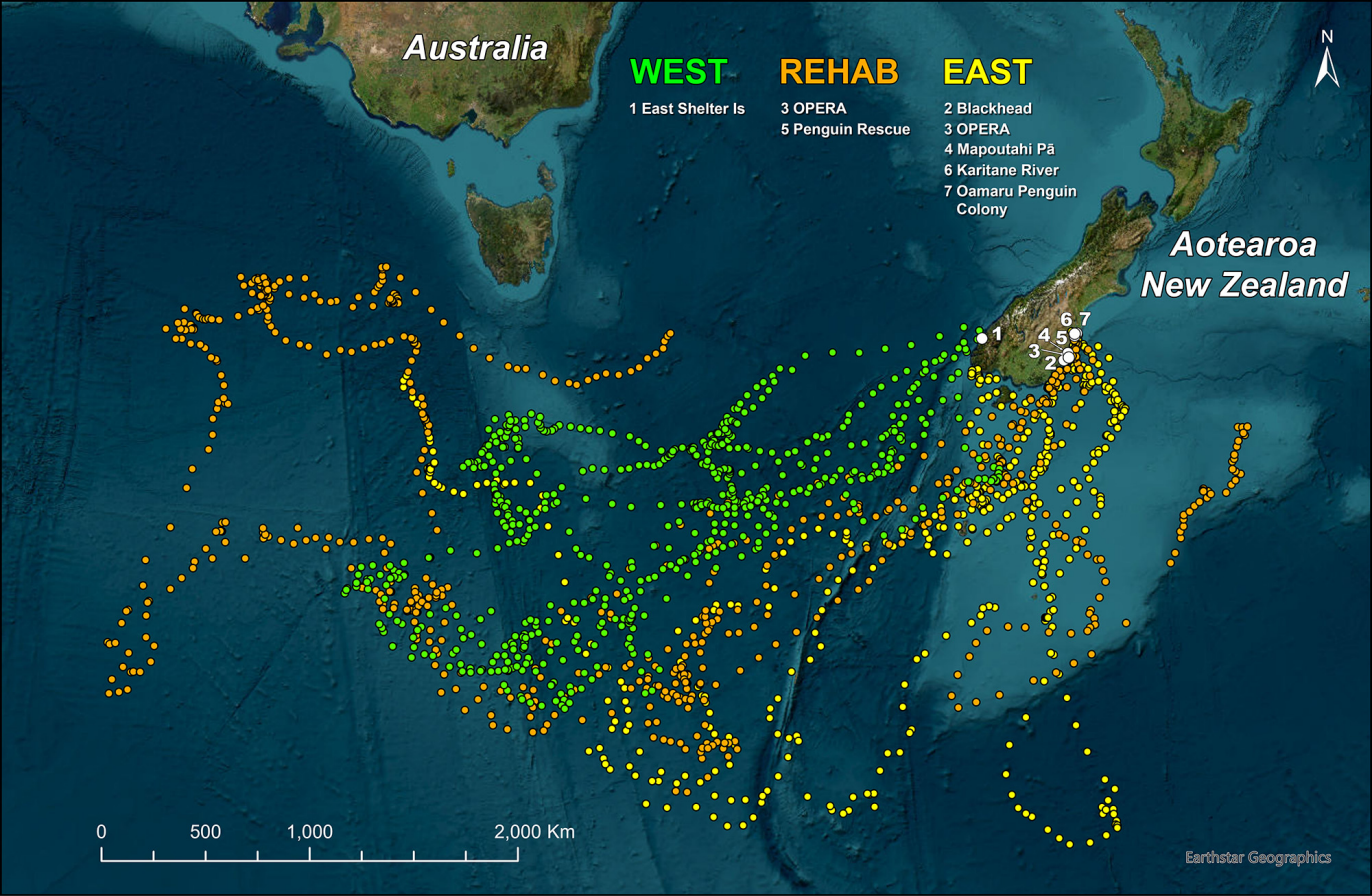
Overview of tawaki deployment locations and satellite tracks. Birds that moulted in the wild or unassisted in care are grouped into West Coast (one site, six birds) and East Coast (five sites, six birds). Penguins that were fed through the moult at rehabilitation centres (two sites, six birds) form the category “Rehab”. Legend provides the colour scheme of the three groups which correspond to the satellite data of birds from the respective group (green = West, yellow = East, orange = Rehab). Numbered white circles indicate location of the different release sites with corresponding names provided below each group header.

For the west coast group, we deployed satellite transmitters on six birds (four females, two males) at East Shelter Island, located at the entrance to Patea/Doubtful Sound, one of Fiordland’s largest breeding sites with an estimated 120–150 breeding pairs (Fig. 1; Mattern, 2013).

For the east coast group, moulters were identified through public and conservation worker reports. Four individuals were accessible for tagging: a female in a rock crevice below the Oamaru Blue Penguin Colony, a male in a small cave along the Karitane River, a female on the foreshore near Mapoutahi Pā north of Dunedin, and a female at Blackhead south of Dunedin (Fig. 1). To complete the group of six, we included two birds (one male, one female; sex determined as described below) uplifted from public sites and transferred to rehabilitation facilities due to human disturbance and the risk posed by uncontrolled dogs. As neither bird was fed during the moult, their physiological condition at departure is considered comparable to wild east coast moulters, despite the atypical moulting location. As such, both birds are retained within the east coast group throughout, *i.e.*, classified by feeding status rather than by uplift history.

The supplementarily fed group consisted of six penguins. Three (one male, two females) moulted at Penguin Place on the Otago Peninsula (now operating as The OPERA), while the other three (all males) were housed at Penguin Rescue, Karitane Lighthouse, North Otago (Fig. 1). Both sites are public-facing wildlife rehabilitation facilities that care for injured, distressed, or vulnerable seabirds, including moulting penguins found in areas accessible to the public or otherwise considered unsafe. Birds are typically admitted following reports from members of the public or conservation staff and are held until the moult is complete and the bird is fit for release. Unlike the east-coast translocations, all birds in this group were hand-fed throughout the moult and are referred to as ‘supplementarily fed birds’ hereafter to distinguish them from unfed birds in the east-coast group which also spent time in rehabilitation. Our three-group design allows the effects of geographic moulting location and supplementary feeding during rehabilitation to be examined independently.

### Satellite transmitter deployments

Birds were tracked using Argos satellite transmitters (SPOT-275 Platform Transmitter Terminals, Wildlife Computers, Redmond, WA, USA; dimensions W × L × H: 15 × 85 × 17 mm; weight: 40 g). Devices were programmed to transmit to Argos satellites up to 15 times per hour. This rate was calibrated to balance data acquisition with battery life, which was expected to last up to six months—sufficient to cover the entire migration period (March–August). The satellite tags were configured to activate only once the saltwater switch registered immersion, *i.e.*, after the penguins had completed their moult and departed to sea at the start of the winter migration.

For deployment, birds were captured by hand from burrows or rock crevices they moulted in and transferred into a cloth bag. Individuals were then weighed using a 5 kg Pesola spring balance to the nearest 50 g, and sex was determined using morphometric measurements following established field sexing criteria for the species (White et al., 2021). Transmitters were fitted using the Tesa-tape method outlined by Wilson et al. (1997). Before attachment, a thin layer of Pattex rubber adhesive (Henkel AG & Co. KGaA, Düsseldorf, Germany) was applied to the feathers beneath the base of each unit, which was then secured with Tesa tape. For additional security, two cable ties were threaded under the device base and tightened with a mechanical cable tie gun, which simultaneously cut the excess strap flush with the locking head; this modification was intended to reduce the risk of birds dislodging the device by working on the tape layer. To further reinforce the attachment, the tape was coated with Araldite rapid-setting epoxy (Selleys, Auckland, New Zealand). The entire handling procedure, from capture to release, took approximately 10–15 min. During this period, birds were kept in cloth bags that covered their heads. West coast and east coast birds were released back into the burrows or sheltered sites where they had been found moulting, while rehabilitation birds (inclusive of fed and unfed birds) were returned to their holding pens prior to release.

Device deployments on East Shelter Islands (west coast group) were conducted on 17 and 18 February 2020. Deployments for the east coast group of birds were more temporally spread out and occurred between 1 February and 9 March 2020. Birds from the supplementarily fed group were fitted with transmitters on the day of their designated releases between 1–27 February 2020.

No bird returned to shore after initial departure; all individuals showed continuous outbound movement from first transmission onward.

### Satellite data analysis

Using Argos settings described above, around 10 positions per bird per day were typically obtained. Because Argos location estimates vary in accuracy, we excluded data points where error could not be calculated (location classes A, B, Z) or where error was >1,500 m (class 0) (c.f. Thomson et al., 2017). The remaining positions were further processed in R using the ‘sdafilter’ function in the *argosfilter* package (Freitas et al., 2008; Freitas, 2022), using a maximum swimming speed threshold of two m/s consistent with Mattern et al. (2025). To account for variation in position fix density among individuals—which ranged from a mean of 2.1 to 7.8 fixes per day—filtered positions were subsequently averaged to one position per bird per UTC day prior to all spatial analyses. This equalises each individual’s temporal contribution to group-level space-use estimates regardless of satellite coverage.

### Data analysis

#### Basic trip parameters

Trip parameters were calculated from filtered positions. Total distance travelled was computed as the sum of straight-line distances between consecutive positions. Maximum distance from the deployment site was the greatest straight-line distance recorded between the deployment location and any position during the tracking period. Mean travel speed was derived by dividing total distance travelled by transmission duration in days.

#### One-way analyses of variance (ANOVAs)

All data were processed in R version 4.5.1 (R Core Team, 2025). One-way analyses of variance (ANOVAs) were run to investigate differences between groups (west coast, east coast and supplementarily fed) in maximum distance from deployment site (km), mean travel speed (km/day), and body mass (g). Group sample sizes were unequal (west coast *n* = 6, east coast *n* = 5, supplementarily fed *n* = 6) owing to the exclusion of one east coast bird that ceased transmitting shortly after deployment. A Tukey HSD *post-hoc* test was run on any significant results and box and whisker plots were produced for each variable.

#### Kernel utilisation distributions

Daily-averaged satellite positions were used to produce 95%, 75%, and 50% kernel utilisation distributions (KUDs) for each group in R. Prior to analysis, all positions were reprojected to a Lambert Azimuthal Equal Area (LAEA) projection centred on the data centroid (155.32°E, 50.32°S) to avoid coordinate distortion near the antimeridian that affected kernel estimation in global projections. Bandwidth estimation was performed separately for each group using the multivariate plug-in estimator ‘Hpi’ from the *ks* package (Duong et al., 2025), applied to kilometre-scaled coordinates for numerical stability. The resulting bandwidth matrix was reduced to an isotropic scalar bandwidth using:

\begin{eqnarray*}h=\mid H{\mid }^{1/4} \end{eqnarray*}

where H is the 2 × 2 plug-in bandwidth matrix returned by ‘Hpi’ and —H— denotes its determinant. This scalar represents the geometric mean spread of the bandwidth matrix across both spatial dimensions and was passed to the ‘kernelUD’ function in the *adehabitatHR* package (Calenge, 2006) as an isotropic smoothing parameter. KUDs were computed on a shared spatial grid at 10 km resolution extending 2,000 km beyond the data extent on all sides, ensuring density surfaces were computed on identical coordinates and were directly comparable across groups. KUD polygons were exported as shapefiles and land areas were removed using the ’st_difference’ function in the *sf* package (Pebesma, 2018).

#### General linear models

General linear models (GLM) were used to investigate whether body mass, sex, group, or some combination of these variables best explained the maximum displacement from the deployment location. All additive and full interaction combinations of these three predictors were fitted using Gaussian distributions, and model performance was compared using AICc values to identify the most parsimonious explanation of variation in displacement.

#### Permits and animal ethics

The study was conducted in accordance with relevant national, international (Field et al., 2019), and institutional animal care guidelines. It operated under a research permit (38882-RES) granted by the Department of Conservation under the New Zealand Wildlife Act 1953, and all procedures received approval from the University of Otago Ethics Committee (D69/17).

## Results

Satellite transmitters were deployed on 18 tawaki between 1 February and 11 March 2020. One east coast bird (device id: 197054, Table 1) ceased transmitting after 3.7 days and was excluded from all spatial and movement analyses, though retained for body mass comparisons, yielding a final sample of 17 birds for movement analyses (west coast—*n* = 6, east coast—*n* = 5, supplementarily fed—*n* = 6, Fig. 1). All other birds could be tracked until they reached the maximum distance from the place of origin so that the outward bound section of the migration was recorded for all birds. Complete return migrations were recorded for one west coast bird and two east coast birds; no supplementarily fed bird was tracked returning to the mainland. Transmission durations ranged from 49.3 to 181.2 days across all groups. Mean transmission duration was 104.7 days for west coast birds (range: 61.5–158.6 days), 89.4 days for east coast birds (range: 49.3–113.8 days), and 113.1 days for supplementarily fed birds (range: 50.0–181.2 days); individual transmission durations are provided in Table 1.

**Table 1:** Bird information. Trip statistics from 18 tawaki tracked throughout the study (east coast - *n* = 6, west coast - *n* = 6, supplementarily fed - “Sup- Fed”, *n* = 6). One east coast bird (Sat id: 197054) ceased transmitting after 3.7 days and was excluded from all spatial and movement analyses, though retained for body mass comparisons, yielding a final sample of 17 birds for movement analyses (west coast - *n* = 6, east coast - *n* = 5, supplementarily fed - “Sup- Fed”, *n* = 6).

**Group**	**Name**	**Deployment site**	**Sat ID**	**Sex**	**Weight (g)**	**Date of first transmission**	**Transmission duration (days)**	**Distance travelled (km)**	**Average travel speed (km/day)**	**Max distance from origin (km)**	**Trip completed**
East Coast	Tom	Kakanui River	197044	M	2,350	11/03/2020	102.0	7,407.4	72.7	2,442.3	Yes
East Coast	Eckart	Penguin Place	197048	M	3,000	22/02/2020	49.3	4,841.7	98.2	2,997.4	No
East Coast	Tinu	Oamaru Blue Penguin Colony	197052	F	2,200	02/02/2020	85.0	5,700.9	67.1	3,069.8	No
East Coast	Rita	Oamaru Blue Penguin Colony	197053	F	2,200	16/02/2020	97.1	5,668.9	58.4	1,215.5	Yes
East Coast	Paloma	Doctor’s Point	197043	F	2,300	15/02/2020	113.8	7,967.1	70.0	3,200.4	No
East Coast	Pitti	Blackhead	197054	F	2,400	24/02/2020	3.7	254.9	69.2	223.3	No
Sup- Fed	Jean	Penguin Place	197047	F	3,200	28/02/2020	96.1	5,725.8	59.6	1,895.8	No
Sup- Fed	Maurice	Katiki Point	197045	M	4,200	24/02/2020	181.2	12,948.4	71.5	4,025.8	No
Sup- Fed	Harper	Penguin Place	197049	F	3,100	13/02/2020	99.1	6,857.2	69.2	4,260.4	No
Sup- Fed	Anton	Penguin Place	197050	M	3,400	14/02/2020	50.0	5,230	104.5	2,962.8	No
Sup- Fed	Tony	Penguin Place	197051	M	3,100	16/02/2020	99.0	7,195.7	72.7	2,683.1	No
Sup- Fed	Fridolin	Katiki Point	197046	M	4,100	16/02/2020	153.2	12,128.1	79.2	4,945	No
West Coast	Giovanni	East Shelter Island	197042	M	2,750	21/02/2020	102.5	7,895.8	77.1	3,301.9	No
West Coast	Ipsis	East Shelter Island	197041	F	2,700	27/02/2020	63.0	4,965.6	78.8	2,836.4	No
West Coast	Rebecca	East Shelter Island	197040	F	2,150	22/02/2020	106.4	8,083	76.0	2,585	No
West Coast	K-Tec II	East Shelter Island	197039	F	2,450	19/02/2020	134.9	8,743.2	64.8	2,849	No
West Coast	Guido	East Shelter Island	197038	M	3,050	29/02/2020	61.5	4,927.1	80.1	3,158.8	No
West Coast	Blubi	East Shelter Island	197037	F	2,300	18/02/2020	158.6	8,375.7	52.8	2,480.9	Yes

### Body mass

**Figure 2:**
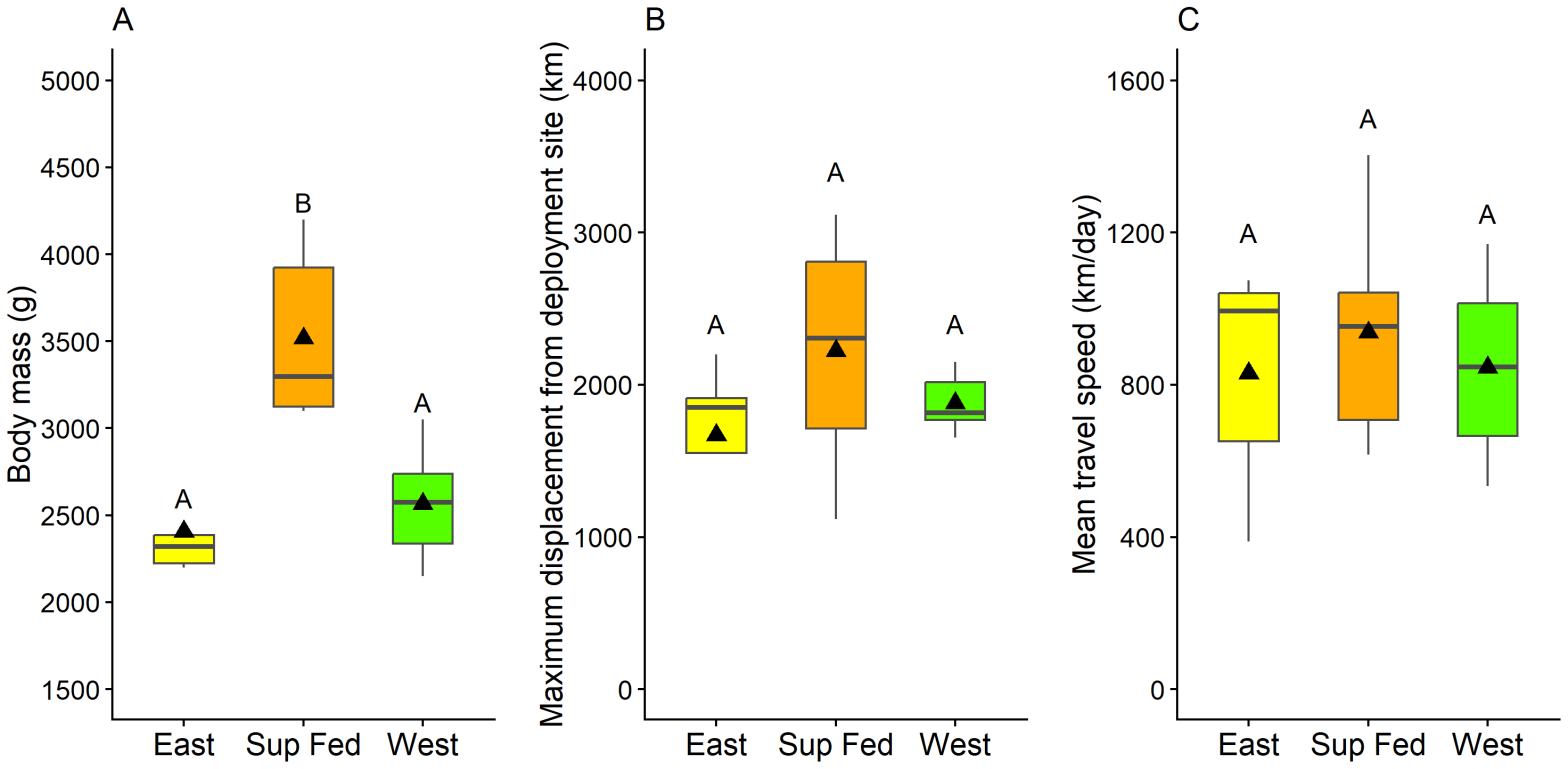
Boxplots of body mass (g), maximum displacement from deployment location (km), mean travel speed (km/day). Box and whisker plots showing post-moult tawaki (A) body mass (g) prior to their feeding journeys, (B) maximum displacement from deployment location (km) during their feeding journeys, (C) mean travel speed (km/day) during their feeding journeys from three colonies, east coast (“East”, *n* = 6 for (A), *n* = 5 for (B) and (C)), west coast (“West”, *n* = 6), and supplementarily fed (“Sup Fed”, *n* = 6). Triangles show mean (A) - body mass (g), (B) - maximum displacement from deployment location (km), (C) - mean travel speed (km/day). Letters over boxes indicate significant difference between groups from Tukey HSD tests.

Body mass differed significantly among the three groups at the start of their post-moult migration (one-way ANOVA, *F*_2_ = 14.29, *p* < 0.001; Fig. 2A). Supplementarily fed birds were substantially heavier than both west coast birds (Tukey HSD, *p* = 0.002) and east coast birds (Tukey HSD, *p* < 0.001). Body mass did not differ significantly between west coast and east coast birds (Tukey HSD, *p* = 0.737; Fig. 2A).

### Migratory trajectories and space use

**Figure 3:**
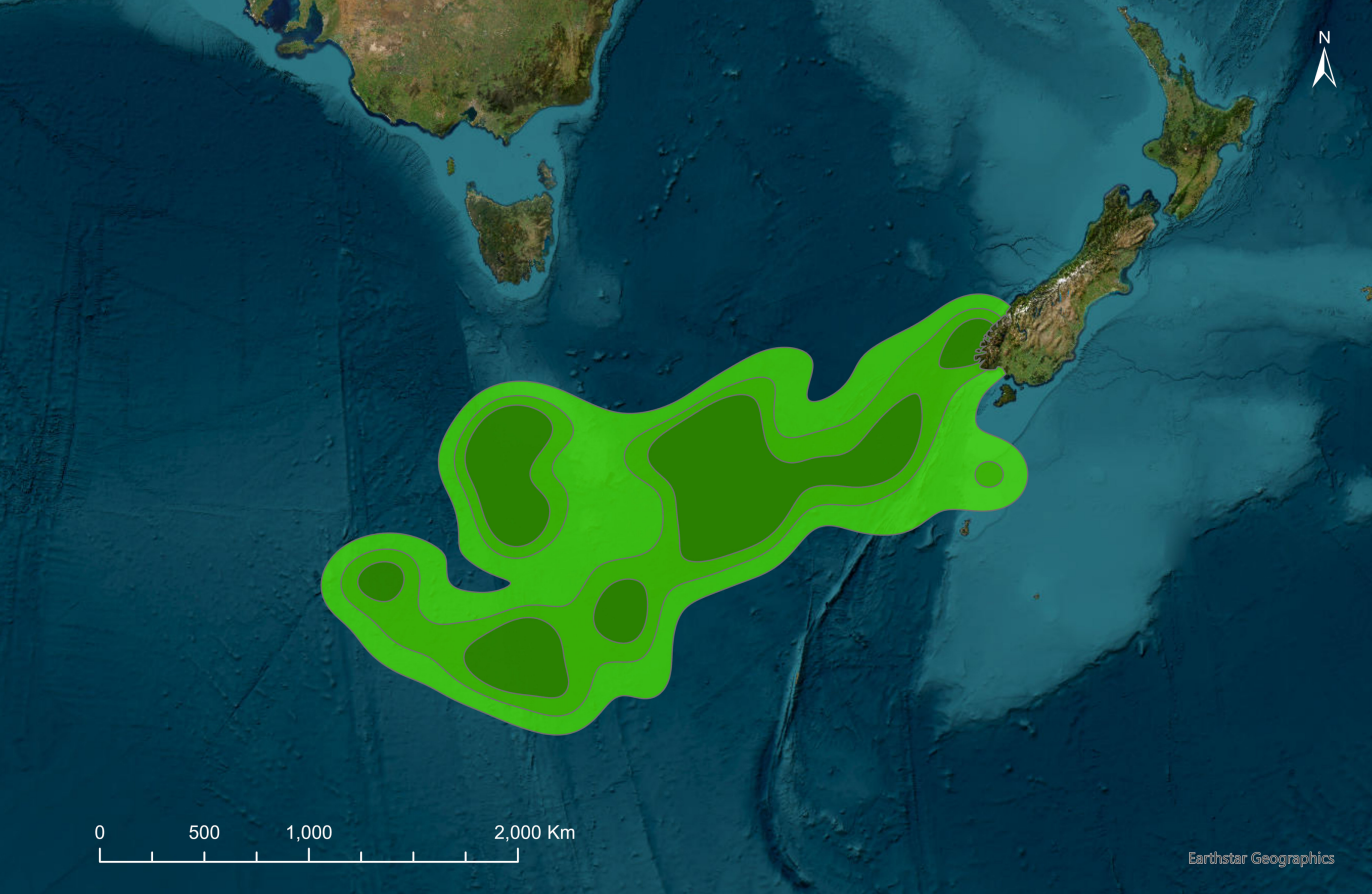
West coast tawaki kernel utilisation distribution. Kernel utilisation distribution calculated from satellite transmitter deployments on west coast birds (*n* = 6). Colours represent 95% (light green), 75% (mid-green), 50% (dark green) kernel utilisation distributions.

West coast birds departed from Fiordland on a south-westerly trajectory, consistent with previous tracking studies, ultimately reaching the Polar Frontal Zone (PFZ) south of Australia (Figs. 1 and 3). Their 95% KUD encompassed 1.38 million km^2^, with a core range (50% KUD) of 402,000 km^2^ (Table 2).

**Figure 4:**
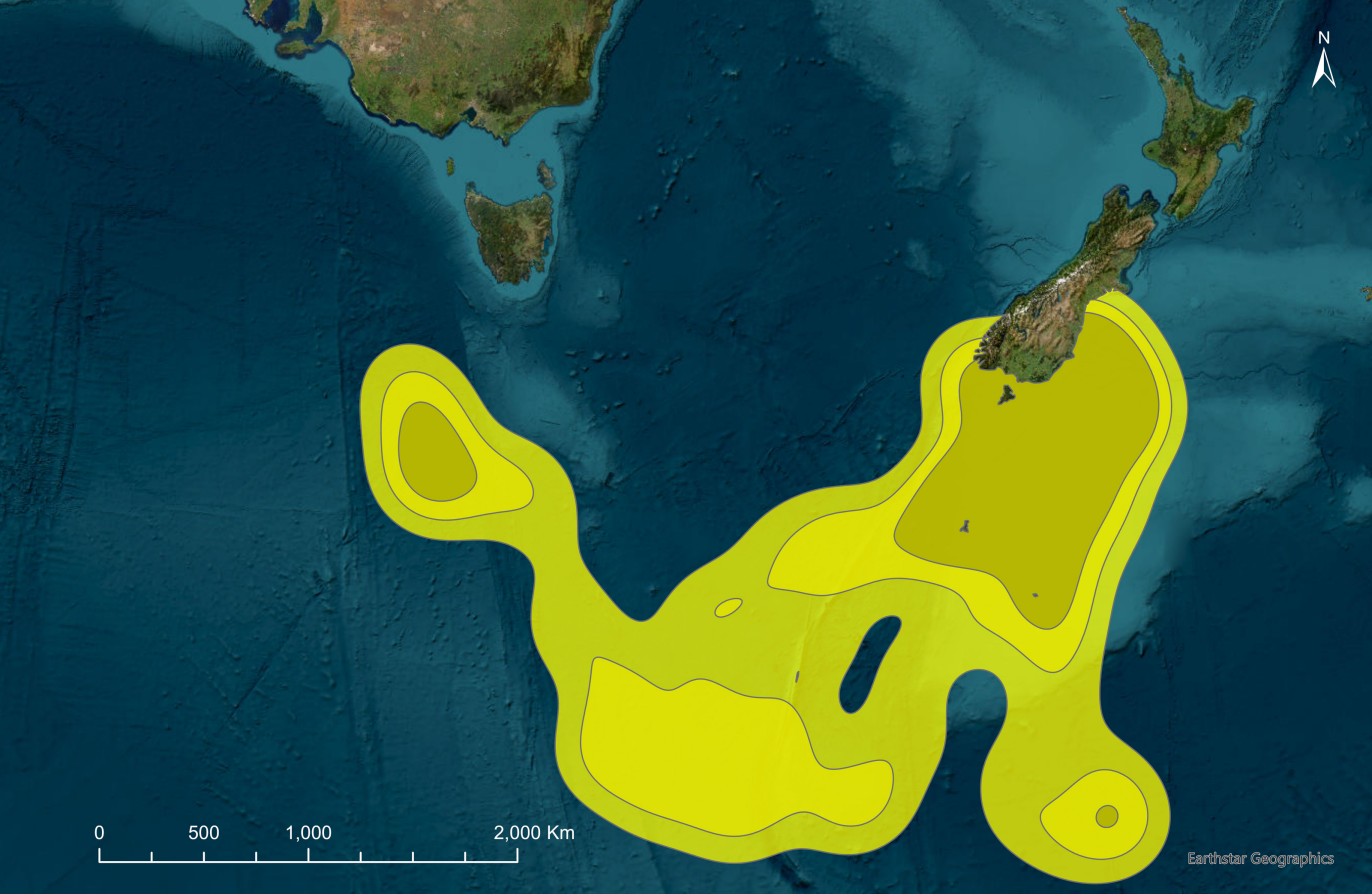
East coast tawaki kernel utilisation distributions. Kernel utilisation distribution calculated from satellite transmitter deployments on east coast birds (*n* = 5). Colours represent 95% (light yellow), 75% (mid-yellow), 50% (dark yellow) kernel utilisation distributions.

East coast birds migrated predominantly southward and south-westward, accessing the PFZ directly south of mainland New Zealand, though some individuals followed trajectories that converged with those of west coast birds (Figs. 1 and 4). The east coast 95% KUD (2.13 million km^2^) was larger than that of west coast birds, and their core range (50% KUD; 490,000 km^2^) was comparable in size but spatially distinct, with negligible overlap with the west coast core range (11 km^2^; Table 3). The 95% KUDs of the west and east coast groups overlapped by 501,468 km^2^, representing modest shared use of the broader migratory corridor.

**Figure 5:**
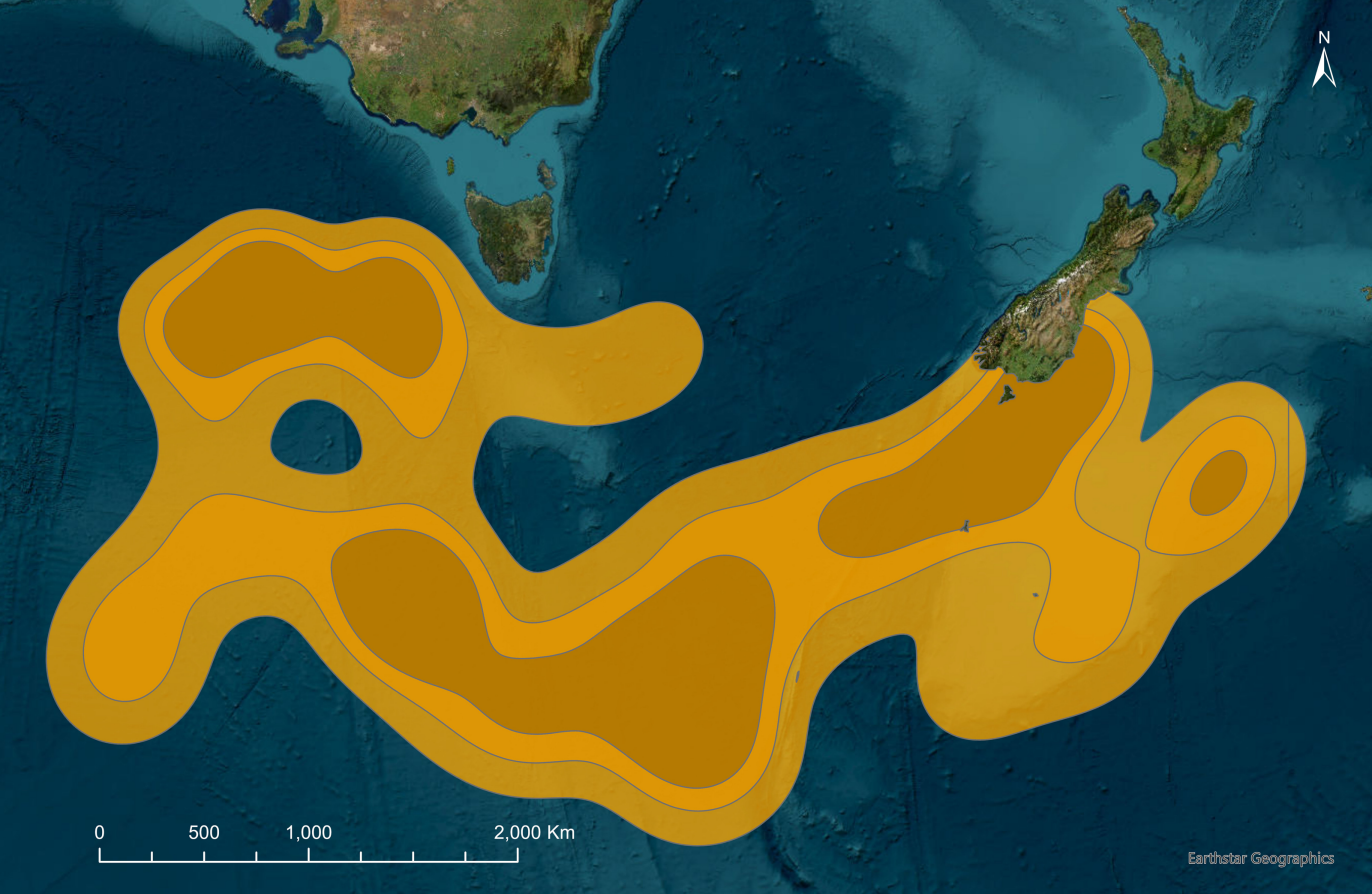
Supplementarily fed birds kernal utilisation distribution. Kernel utilisation distribution calculated from satellite transmitter deployments on supplementarily fed birds (*n* = 6). Colours represent 95% (light orange), 75% (mid-orange), 50% (dark orange) kernel utilisation distributions.

**Figure 6:**
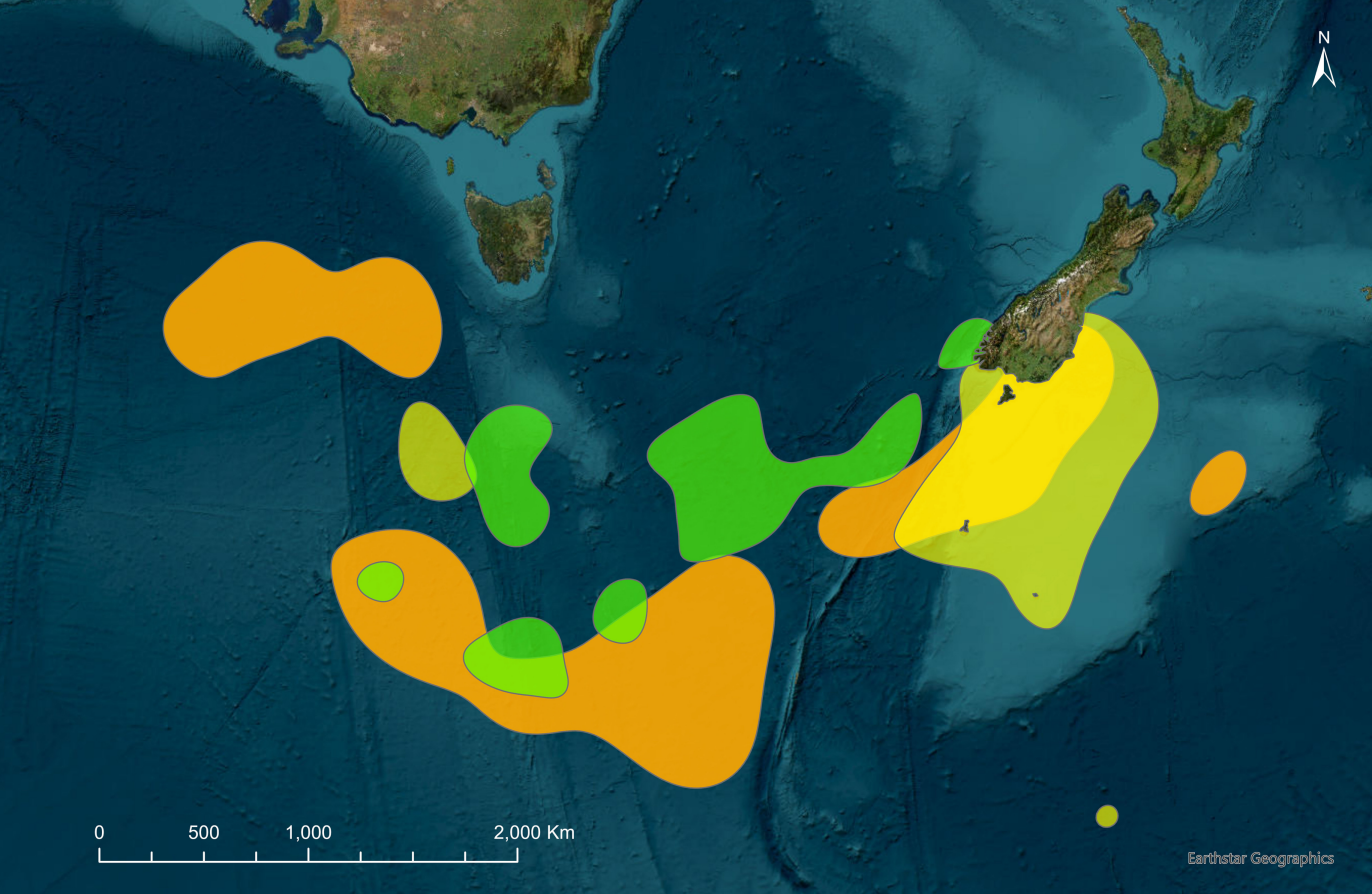
50% kernel utilisation distribution overlap between groups. 50% kernel utilisation distribution calculated from satellite transmitter deployments on supplementarily fed birds (orange; *n* = 6), west coast birds (green; *n* = 6), and east coast birds (yellow; *n* = 5).

Supplementarily fed birds migrated south-westward along a trajectory broadly similar to west coast birds but ranged far more widely. Their 95% KUD was 3.96 million km^2^—roughly three times that of west coast birds and nearly twice that of east coast birds—and a core range of 1.07 million km^2^ (Table 3; Figs. 1 and 5). Despite this broader distribution, their core range overlapped more extensively with east coast birds (225,954 km^2^) than with west coast birds (70,739 km^2^) (Fig. 6, Table 3), and the same pattern held at the 95% KUD level (Table 3).

### Migratory displacement and travel speed

Supplementary fed birds reached the greatest maximum displacement from their deployment sites, followed by west coast and then east coast birds, though this pattern was not statistically significant (one-way ANOVA, *F*_2_ = 1.384, *p* = 0.283; Fig. 2B). Mean travel speed was similarly consistent across groups (one-way ANOVA, *F*_2_ = 0.294, *p* = 0.783; Fig. 2C).

Body mass alone is the most parsimonious predictor of maximum displacement from the deployment site (AICc = 262.660; Table 4), with a positive, statistically well-supported relationship between departure mass and displacement (*β* = 0.565, SE = 0.183, *p* = 0.008; Table 4). All competing models were at least 3.4 AICc units behind, indicating considerable support for the weight-only model over any alternative. Notably, all models incorporating group membership performed substantially worse (ΔAICc ≥ 6.4), indicating that group affiliation contributed no independent explanatory power once body mass was accounted for.

## Discussion

Wild tawaki from both coasts followed broadly similar post-moult migratory trajectories, offset laterally in a manner consistent with their respective moulting locations. West coast birds tracked southwest toward the Polar Frontal Zone south of Tasmania, and east coast birds headed more directly south toward comparable habitat south of the New Zealand mainland. Against this backdrop of geographic predictability, the behaviour of supplementarily fed birds stands out. Despite moulting on the east coast, they ranged far more widely than either wild group, with individual trajectories spanning the space between the two wild groups and beyond. These results suggest that tawaki post-moult migration reflects a broadly consistent directional tendency modulated by moulting location, but that this pattern breaks down when birds depart in an artificially elevated physiological state.

### Migratory trajectories reflect moulting location

West coast birds departed Fiordland on a southwest trajectory toward the Polar Frontal Zone (PFZ) south of Tasmania, consistent with both the pre-moult dispersal patterns described by Mattern et al. (2018) and the winter migration routes documented by Mattern et al. (2025). East coast birds, by contrast, showed a split pattern. Some individuals followed a broadly southward trajectory, accessing the PFZ directly south of the New Zealand mainland, a region identified by Mattern et al. (2025) as offering suitable habitat conditions, while others travelled southwest toward the same destination as west coast birds. This divergence within the east coast group is striking and suggests that moulting location alone is not sufficient to determine migratory direction. While geographic starting point likely influences the initial bearing of departure, some east coast birds appear to override any local directional bias and converge on the southwest trajectory characteristic of west coast populations.

**Table 2:** Kernel utilisation density area. Area (km^2^) of 95%, 75%, and 50% kernel utilisation distributions calculated from satellite transmitter deployments from three tawaki colonies, west coast (*n* = 6), east coast (*n* = 5), and supplementarily fed (*n* = 6).

	**West**	**East**	**Supplementarily fed**
Area 50%	401,598	490,090	1,069,634
Area 75%	773,160	1,104,676	2,157,525
Area 95%	1,377,698	2,132,575	3,955,944

**Table 3:** Kernel utilisation density overlap. Area overlap (km^2^) of kernel utilisation distributions (KUD) calculated from satellite transmitter deployments from three tawaki colonies, west coast (*n* = 6), east coast (*n* = 5), and supplementarily fed (“Sup. Fed”, *n* = 6).

	**50% KUD overlap**		**75% KUD overlap**		**95% KUD overlap**	
	East	Sup. Fed	East	Sup. Fed	East	Sup. Fed
West	11	70,739	54,362	348,242	501,467	915,687
East	–	225,954	–	641,056	–	1,578,212

One possible explanation for this within-group variation is the life-history stage. Tawaki that are committed breeders returning to west coast colonies may be more focussed on efficiency. They may want to reach the most productive foraging grounds quickly, therefore, favouring the well-established southwest corridor. Non-breeding or younger individuals, freed from the energetic demands of a subsequent breeding attempt, may have greater latitude to exploit closer, if less productive, foraging habitat to the south and southeast. Mattern et al. (2025) noted that the Campbell Plateau region southeast of New Zealand contains only a limited band of suitable habitat, suggesting it may be adequate for birds with lower energetic requirements but suboptimal for birds needing to return to breed in good condition. This interpretation is consistent with observations in other long-lived seabirds. Orgeret et al. (2019) found that juvenile king penguins (*Aptenodytes patagonicus*) undertake more exploratory and extended first migrations than non-breeding adults. For juvenile king penguins, initial migratory routes appear to be governed more by innate tendency than by social learning (Orgeret et al., 2019). A similar age- or stage-related divergence in tawaki cannot be confirmed from the present data, as the breeding status and age of tagged individuals were unknown.

The role of genetic predisposition in structuring tawaki migration deserves consideration as it has been shown to affect migration in passerine birds (Helbig, 1991). Mattern et al. (2018) raised the possibility that the consistent southwest corridor used by west coast birds reflects a deeply conserved migratory tendency, potentially rooted in the subantarctic evolutionary origins of crested penguins (Cole et al., 2019). The fact that most rehabilitated east coast birds, presumably originating from west coast colonies given the species’ breeding distribution, also followed a southwest trajectory lends some support to this interpretation. However, the divergent trajectories of some wild east coast birds, which adopted a more southward route, indicate that this tendency is not immutable and may be modulated by individual state or experience. Tawaki migration therefore appears to reflect not a simple genetic programme nor pure environmental responsiveness, but an interaction of inherited directional tendency with individual condition and, potentially, life-history stage. Whatever consistency exists between wild populations, however, is largely overridden in penguins that receive supplementary feeding in rehabilitation during the moult which fundamentally alters the physiological state at which birds enter the migration.

### Body mass and the consequences of supplementary feeding

Supplementarily fed birds were significantly heavier at the start of their post-moult migration than birds in either wild group, a direct consequence of supplementary feeding throughout the moult. West and east coast birds did not differ significantly in body mass, confirming that moulting location, rather than feeding regime, was the key variable distinguishing these groups. Body mass emerged as the most parsimonious predictor of maximum displacement in our GLM, with a statistically well-supported positive relationship between mass and displacement. Notably, group membership added no independent explanatory power once body mass was accounted for, suggesting that the migratory differences observed among groups are mediated through departure body condition rather than reflecting a direct effect of group identity. Heavier birds carry larger energy reserves at departure, and it is during the early outbound phase of migration—before significant foraging has occurred—that this difference is most likely to manifest behaviourally. Rather than being channelled efficiently toward productive foraging grounds by genuine energetic need, supplementarily fed birds may lack the urgency that drives wild birds to orient directly toward the most rewarding habitat. This could cause them to meander into areas of lower productivity, where unsuccessful foraging then forces further searching, compounding the initial directional error and ultimately resulting in the far more widespread distribution observed in this group.

**Table 4:** AICc model ranking. Model selection results from general linear models predicting maximum displacement from the deployment site (km) in post-moult tawaki (*n* = 17). All additive and interaction combinations of body mass (g), sex, and group (west coast, east coast, supplementarily fed) were fitted using Gaussian distributions and ranked by Akaike information criterion (AICc). ΔAICc is the difference in AICc relative to the top-ranked model.

**Model**	**AICc**	**ΔAICc**
Weight	262.66	0
Weight+Sex	266.05	3.39
Weight*Sex	268.48	5.82
Weight+Group	269.06	6.4
Weight+Sex+Group	269.69	7.03
Weight*Group	271.42	8.76
Sex	273.99	11.33
Group	274.28	11.62
Sex+Group	284.01	21.35
Sex*Group	286.72	24.06
Weight*Group*Sex	392.92	130.26

The space-use data support this interpretation. Supplementarily fed birds’ 95% KUD was roughly two to three times that of either wild group. Furthermore, their core range showed more overlap with east coast birds than with west coast birds despite the majority of individuals following a broadly southwest trajectory. This combination of a familiar general bearing with unusually wide dispersion is consistent with birds that departed with their directional tendency intact but without the energetic imperative that likely keeps wild birds on a focused and efficient migration.

Some supplementary fed birds also spent longer at sea before returning to the mainland, raising the possibility that they arrived at breeding sites after pair bonds had been established and nesting had begun, missing the breeding season as a result. This need not, however, reflect a rehabilitation effect alone: birds that were pre-breeders would have no imperative to return on a breeding schedule regardless of their moulting history. Without knowledge of the age or breeding status of individual study birds, these two explanations cannot be distinguished, and both remain speculative. Nevertheless, given the demonstrable effect of supplementary feeding on body mass and the downstream consequences for movement patterns documented here, the possibility that supplemental feeding during rehabilitation alters the timing of return and therefore breeding participation cannot be dismissed.

### Implications for rehabilitation practice

The annual moult in penguins is an energetically self-contained process. Birds fast on land, drawing on body reserves accumulated prior to moult, and depart once feather replacement is complete (Garcia Borboroglu & Boersma, 2013). Supplementary feeding during this period inflates body mass beyond what birds would achieve naturally, and the consequences documented here—wider ranging movements, probable entry into suboptimal foraging habitat, and potentially delayed return to breeding sites—suggest that this intervention, however well intentioned, carries real costs.

It is worth emphasising that this finding does not diminish the value of rehabilitation. Uplifting moulting birds from sites where they face predation from dogs, cats, and mustelids, or direct human disturbance, remains a justified and important intervention. The key refinement is to allow the moult itself to proceed as naturally as possible once a bird is in a safe holding environment, and without supplementary feeding. Decisions to provide supplementary feed are often based on simple body mass thresholds, but this approach is problematic for several reasons. In well-studied species such as yellow-eyed penguins (*Megadyptes antipodes*), sufficient data exist to define meaningful condition benchmarks, but for crested penguins, including tawaki, no such thresholds have been established. Even where they have, a single mass threshold fails to account for the considerable natural variation between individuals: a small male and a large female may sit on opposite sides of the same cutoff for entirely natural reasons. Visual and behavioural assessment likely provides a more reliable indicator of genuine poor condition. A bird that appears gaunt with a visible keel toward the end of the moult is exhibiting a normal fasting response; the same appearance early in the moult, before feather loss has begun, is cause for concern. Similarly, lethargy and reduced responsiveness are meaningful signs that a bird is not coping, in a way that body mass alone cannot capture.

Rehabilitation centres should develop feeding protocols that reflect this nuance, rather than applying blanket thresholds that may lead to unnecessary intervention. While visual and behavioural assessment provides valuable indicators of condition, interpreting these cues reliably requires considerable experience with the species. Weight-based targets therefore offer clearer and more objective goals for rehabilitation practice. Based on body mass data from wild birds in this study (Table S1), we propose the following as a preliminary, evidence-based starting point for refining feeding decisions during rehabilitation

(1) that supplementary feeding should not be considered necessary unless a moulting bird fall below the minimum weights recorded in wild birds in this study, that is 2,300 g for males and 2,100 g for females;
(2) target release weights at the end of the moult of approximately 2,900 g for males and 2,300 g for females, not exceeding 3,000 g and 2,700 g respectively.

The lower bounds indicate where intervention may be justified, while the upper bounds reflect the maximum body mass of wild birds at the onset of migration and should not be exceeded. These targets are intended as a provisional reference point rather than a definitive protocol; given the small sample underlying this study, they should be revisited as further data on tawaki body condition and post-release outcomes become available.

Body mass benchmarks of this kind may also vary between years depending on broader environmental conditions. Wild birds tracked in this study during winter 2020, when the Southern Oscillation Index indicated ENSO-neutral conditions (Bureau of Meteorology), were notably lighter at departure (mean 2.65 kg, west and east coast combined) than the wild birds tracked by Mattern et al. (2025) during winter 2019 (mean 3.1 ± 0.4 kg), a year in which a consistently negative Southern Oscillation Index from February through December pointed to weak El Niño-leaning conditions. While we cannot establish a causal link between ENSO phase and pre-moult body condition from these two years alone, the difference illustrates that the wild-bird mass values underlying these targets are not necessarily stable across years, and should be interpreted with that context in mind.

### Limitations

The small sample size within each group limits statistical power and means that individual variation may have an outsized influence on group-level patterns. This is an inherent constraint of working with a cryptic, low-density species breeding in remote and largely inaccessible terrain (Mattern & Wilson, 2019), and larger samples are unlikely to be achievable in future studies of this kind. Similarly, the absence of age and breeding status information for tracked individuals is a fundamental limitation rather than a readily addressable one. Tawaki breed in dense forest where chicks often disperse before they can be reliably marked, and no method exists for ageing adults in the field. The effect of life-history stage on migratory patterns remains speculative, and the within-group variation observed in east coast birds cannot be attributed with confidence to differences between breeders and non-breeders.

Incomplete transmission records furthermore mean that total distance travelled was underestimated for individuals that were still at sea when transmission ceased, as the return journey was not recorded for most birds. Mean travel speed may also be affected, though the direction of any bias is uncertain as return travel speed could differ from outbound speed. However, since transmission ended before completion of the return journey across all three groups, any resulting bias is unlikely to systematically inflate between-group differences in these metrics. Maximum displacement is unaffected by this issue, as all 17 birds included in the spatial analysis were tracked until they reached their point of greatest distance from the deployment site.

The unequal sex ratios across groups, combined with small sample sizes, preclude a fully balanced assessment of sex-related differences in body mass or migratory behaviour; potential sex effects cannot be entirely disentangled from group-level patterns in the ANOVA framework used here, though the GLM analysis explicitly tested sex as a predictor and found it added no independent explanatory power (Table 4).

The temporal spread of deployments across groups (1 February to 11 March 2020) means that birds entered the water at different calendar dates, which may have exposed them to different environmental conditions at the start of migration independently of group assignment. While this variation also exists within groups, and therefore cannot systematically account for between-group differences, it remains a potential source of confounding that cannot be fully disentangled from group effects at the available sample sizes.

Nevertheless, the patterns documented here represent a substantial advance on what was previously known, and the three-group design provides the clearest picture yet of how moulting location and rehabilitation interact to shape tawaki post-moult migration. Management decisions regarding the handling of moulting tawaki in rehabilitation can be informed by this evidence, even in the absence of a complete mechanistic understanding.

## Conclusion

This study provides evidence that tawaki post-moult migration is shaped by a combination of moulting location and physiological state at departure, operating against a backdrop of what could well be a broadly conserved directional tendency inherited from subantarctic crested penguin ancestors. Wild birds from both coasts followed broadly southwest trajectories, offset laterally in a manner consistent with their respective moulting locations. Supplementarily fed birds departed with their directional tendency apparently intact, but possibly lacking the energetic imperative that keeps wild birds focused on an efficient migration. The result was a far more dispersed and variable movement pattern with potential consequences for foraging success and breeding participation. These findings advance our understanding of tawaki post-moult migration, and offer an evidence-based reference point for refining rehabilitation practice for this and potentially other penguin species undergoing moult in human care.

##  Supplemental Information

10.7717/peerj.21630/supp-1 Supplemental Information 1 Box plots code R code to create blox plots used in the manuscript

10.7717/peerj.21630/supp-2 Supplemental Information 2 Tawaki GLM and AICc R Code R code embedded with data to replicate GLM and AICc analysis in the manuscript

10.7717/peerj.21630/supp-3 Supplemental Information 3 Kernel density estimation R code for the kernel density estimation done in the manuscript. Data is stored in a separate file.

10.7717/peerj.21630/supp-4 Supplemental Information 4 Mean, maximum, minimum body mass (g) of tawaki in study Mean, maximum and minimum body mass (g) of post-moult tawaki prior to their feeding journeys from three colonies, east coast (*n* = 6), west coast (*n* = 6), and supplementarily fed (*n* = 6) split by sex.

10.7717/peerj.21630/supp-5 Supplemental Information 5 Tawaki daily average Complete data needed for kernel density estimate analysis. Includes all fixes from birds included in the study with latitude and longitude of fix, time and date of fix, sat tag ID, bird ID, group ID, and number of fixes.

## Additional Information and Declarations

### Data Availability

The following information was supplied regarding data availability: The data and code are available in the Supplementary Files.
