# Supplementary material for: Supercharged penguins—supplementary feeding reshapes tawaki/Fiordland penguin winter migration
Source: PeerJ. 2026 Aug 10;14:e21630. doi: 10.7717/peerj.21630 (PMC13471748; doi:10.7717/peerj.21630)
Supplement: Supplementary file 1 [file peerj-14-21630-s004.docx]

|  | **Sex** | **Mean body weight** | **Maximum body weight** | **Minimum body weight** |
| --- | --- | --- | --- | --- |
| **East Coast** | Male | 2675 | 3000 | 2350 |
|  | Female | 2275 | 2400 | 2200 |
| **West Coast** | Male | 2900 | 3050 | 2750 |
|  | Female | 2400 | 2700 | 2150 |
| **Supplementarily Fed** | Male | 3700 | 4200 | 3100 |
|  | Female | 3150 | 3200 | 3100 |
